# Retraction: Utilizing Artificial Intelligence Among Patients With Diabetes: A Systematic Review and Meta-Analysis

**DOI:** 10.7759/cureus.r168

**Published:** 2025-02-05

**Authors:** Abdullah Alhalafi, Saif M Alqahtani, Naif A Alqarni, Amal T Aljuaid, Ghade T Aljaber, Lama M Alshahrani, Hadeel Mushait, Partha​ A Nandi

**Affiliations:** 1 Department of Family and Community Medicine, University of Bisha, Bisha, SAU; 2 College of Medicine, University of Bisha, Bisha, SAU; 3 Department of Medicine, Batterjee Medical College, Aseer, SAU; 4 College of Medicine, King Khalid University, Abha, SAU

This article has been retracted by the Editors-in-Chief. All eight studies are incorrectly presented as randomized controlled trials when they are actually systematic reviews, validation studies or AI modeling studies. The risk ratios and confidence intervals presented in this article could not have been derived from these studies. As a result, this article has been retracted due to validity concerns and data fabrication.

